# May Autogenous Grafts Increase the Effectiveness of Hyalonect Membranes in Intraosseous Defects: An Experimental In Vivo Study

**DOI:** 10.3390/medicina57050430

**Published:** 2021-04-29

**Authors:** Caner Yilmaz, Selim Ersanli, Murat Karabagli, Vakur Olgac, Nilufer Bolukbasi Balcioglu

**Affiliations:** 1 Department of Oral Implantology, Faculty of Dentistry, Istanbul University, Istanbul 34093, Turkey; caneryilmaz3@gmail.com (C.Y.); selimersanli@gmail.com (S.E.); 2 Department of Surgery, Faculty of Veterinary, Istanbul University Cerrahpasa, Istanbul 34098, Turkey; murat.karabagli@iuc.edu.tr; 3 Department of Tumor Pathology, Institute of Oncology, Istanbul University, Istanbul 34093, Turkey; volgac@istanbul.edu.tr

**Keywords:** Hyalonect, hyaluronic acid, hyaluronan, guided bone regeneration, autogenous bone graft, collagen membrane, in vivo model, bone defect

## Abstract

*Background and Objectives*: Guided bone regeneration (GBR) surgeries are used for dental implant placements with insufficient bone volume. Biomaterials used in GBR are expected to produce sufficient volume and quality of bone swiftly. This study aims to histologically evaluate the effectiveness of the use of Hyalonect membranes alone or with autogenous grafts in intraosseous defects. *Materials and Methods*: This study is an experimental study on sheep. Surgeries were performed under general anesthesia in accordance with ethical rules. Five 10 mm defects were surgically created in each ilium of six sheep. One defect was left empty in each ilium (group ED). The defects in the experimental group were covered with Hyalonect membrane while unfilled (group HY) or after being filled with autogenous bone grafts (ABG) (group G+HY). In the control group, the defects were either covered with collagen membrane while unfilled (group CM) or after being filled with the ABG group (G+CM). The sheep were histologically and histomorphometrically evaluated after being postoperatively sacrificed in the third and sixth week (three animals in each interval). *Results*: All animals completed the study without any complications. No difference was found between groups in the third and sixth weeks regarding the inflammation, necrosis, and fibrosis scores. The G+CM (52.83 ± 3.06) group was observed to have a significantly higher new bone formation rate than all the other groups in the third week, followed by the G+HY group (46.33 ± 2.25). Similar values were found for HY and CM groups (35.67 ± 4.55 ve 40.00 ± 3.41, respectively, *p* = 0.185), while the lowest values were observed to be in group ED (19.67 ± 2.73). The highest new bone formation was observed in group G+CM (82.33 ± 4.08) in the sixth week. There was no difference in new bone formation rates between groups G+CM, G+HY (77.17 ± 3.49, *p* = 0.206), and CM (76.50 ± 2.43, *p* = 0.118). The insignificant difference was found ED group and group HY (55.83 ± 4.92, 73.50 ± 3.27, respectively, *p* = 0.09). The residual graft amount in the G+CM group was found to be statistically significant at 3 weeks (*p* = 0.0001), compared to the G+HY group, and insignificantly higher at the 6th week (*p* = 0.4). *Conclusions*: In this study, close values were observed between G+HY and G+CM groups. Further experimental and clinical studies with different graft materials are required to evaluate the effectiveness of HY in GBR.

## 1. Introduction

Sufficient amount of healthy bone is a prerequisite for the treatment of partial or total edentulism by the use of dental implants. Tooth extractions, periodontal disease, trauma, and pathologies cause vertical and horizontal loss in the mandible and maxilla. Longer periods of edentulism tend to result in a greater amount of resorption. Guided bone regeneration (GBR) surgeries are used for the treatment of such losses. Different types (cortical or cancellous) and different sizes (particulate, putty, or block) of graft materials from various sources (autogenous bone, allogenic bone, xenografts, or allogenic bone) are used for GBR operations. The autogenous bone grafts, despite their drawbacks, such as donor-site morbidity, the requirement of extra procedures for its collection, the amount of resorption, and the unpredictability of its duration, are recognized as the benchmark for bone regeneration operations due to their osteogenic, osteoinductive and osteoconductive effects [1,2]. Allogenic grafts, xenografts, or alloplastic grafts are used to eliminate those drawbacks. Membranes need to be used in order to prevent the degradation of faster proliferating epithelial and connective tissues and to stabilize the graft to make bone-making tissues prevalent in the defect area in GBR [3]. Resorbable (such as collagen membrane) or nonresorbable (such as polytetrafluoroethylene (PTFE) or expanded polytetrafluoroethylene membrane (e-PTFE)) membranes are used in maxillofacial surgeries in accordance with the volume and area of the defect and the graft material used. Membranes are expected to have biocompatibility, host compatibility, easy clinical application, sufficient duration of function for regeneration and repair, and space-making ability. PTFE and expanded-polytetrafluoroethylene e-PTFE are the first nonresorbable membranes that were produced. Since these membranes require a second surgery to be removed, and their surfaces have a risk of opening after surgery and that they are relatively difficult to manipulate, resorbable membranes were produced. Collagen-based naturally derived membranes are frequently used as resorbable membranes in GBR. The ideal combination of graft material and membrane has yet to be discovered. The studies focus on biocompatible natural or natural-derived biomaterials, which help to quickly form adequate bone volume and require minimal surgery.

Hyaluronic acid (HA, also named hyaluronate or hyaluronan) is the simplest glycosaminoglycan [4,5]. HA is one of the main components of the hydrophilic polymer and extracellular matrix. It is found in several body fluids, tissues, and organs such as synovial fluid, saliva, serum, gingival crevicular fluid, periodontal tissues, the vitreous humor of the eye, cartilage, brain, muscles, lung, kidney, and brain and reported to have bacteriostatic, anti-inflammatory, fungistatic, antiedematous, osteoinductive, and viscoelastic properties [6,7,8,9,10]. It is effective in the early stages of bone morphogenesis and osteogenesis [11]. It binds to fibrin, fibrinogen, fibronectin, and collagen, which are important for wound healing [12]. Since HA plays role in many biological processes, it is modified with various chemicals to produce mechanically and chemically durable HA-based biomaterials. Today, various forms of HA-based biomaterials are used in the fields of dermatology, orthopedics, ophthalmology, and dentistry.

Hyalonect (Fidia Advanced Biopolymers SRL, Albano Terme, Italy) is a knitted, resorbable, suturable, biocompatible mesh that has been specially developed to provide periost regeneration in orthopedic and traumatic surgeries [13]. Hyalonect is composed of HYAFF 11, a modified hyaluronan fiber. HYAFF 11 is obtained by a complete benzyl-esterification of the carboxyl groups of the D-Glucuronic acid unit of HA to reduce its hydrophilicity and to create solid biomaterial [13,14]. The resorption period is approximately 40 days [14]. One major advantage of this mesh is that during hydrolyzation, it produces a gel substance similar to the natural HA found in the extracellular matrix, and this may result in an increase in cell migration, proliferation, and differentiation of the products that result while Hyalonect degrades [15,16].

To the best of our knowledge, there are no studies in the literature in which autogenous graft and Hyalonect mesh were used together in GBR. The aim of this experimental study is to evaluate histologically and histomorphometrically the effectiveness of using Hyalonect membrane alone or together with autogenous grafts in the treatment of intraosseous defects. At the same time, the data of the experimental groups will be compared with the collagen membrane with/without autogenous bone graft, which is used in GBR in routine clinical applications. Since this study is a preclinical study, an experimental study designed on sheep was planned in order to evaluate the healing in standardized defects in the test and control groups, for the early and late periods and to make the results comparable.

## 2. Materials and Methods

### 2.1. Study Design

This experimental study on six male sheep (mean weight: 54.66 ± 3.55 kg; mean age: 26.33 ± 2.25 months) was conducted.

**Figure 1:**
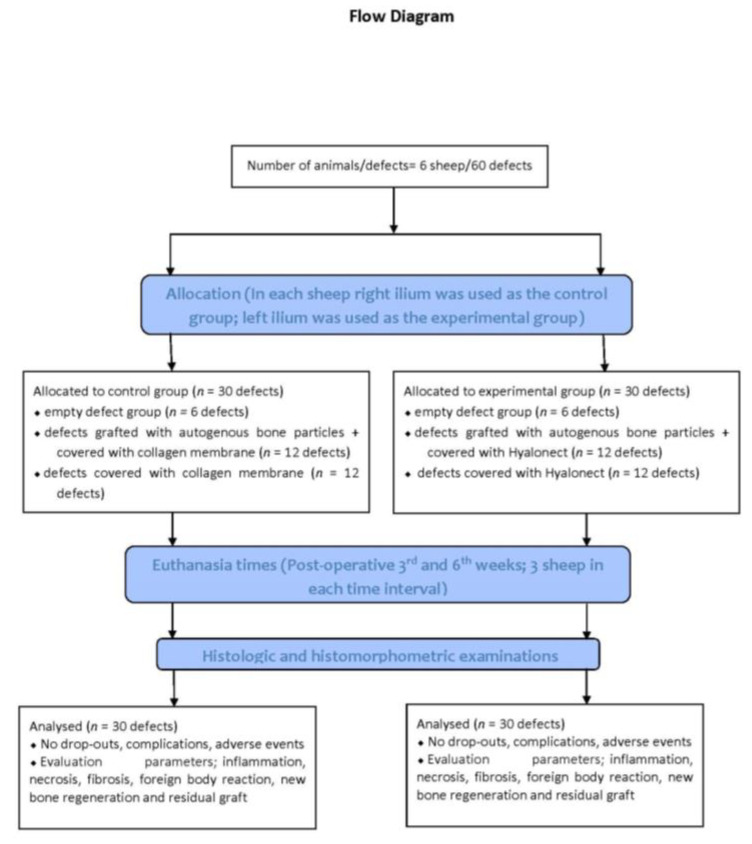
Flow chart of the study.

The study protocol was approved by the Animal Experimentation Ethics Committee of the ˙Istanbul University (approval no: 2018/15; approval date 18 April 2018). All procedures were conducted in accordance with international ethical guidelines for the treatment and welfare of experimental animals. This manuscript was prepared according to the guidelines proposed by the ARRIVE [17] (Figure 1).

### 2.2. Sample Size

The power analysis was performed based on the previous graft healing scores [18] achieved at 0.80 power (StataCorp. 2019. Stata Statistical Software: Release 16. StataCorp LLC, College Station, TX, USA). It was observed that at least five specimens in each group were required at α: 0.05 and power: 0.80. For the study, a total number of 30 defects for the third and sixth week and six specimens for each group were determined.

### 2.3. Surgical Procedures

The animals were fasted 24 h prior to the surgical operation. All animals were administered preanesthetic xylazine (0.2–0.5 mg/kg IM, Rompun, Bayer, Istanbul, Turkey) via vascular access before the surgery. The animals were then intubated following intravenous administration of ketamine hydrochloride (5 mg/kg, Ketalar, Eczacıbası, Istanbul, Turkey). General anesthesia was achieved with 3.5% isoflurane (Forane, Abbott) and sustained with 1.5% isoflurane.

**Figure 2:**
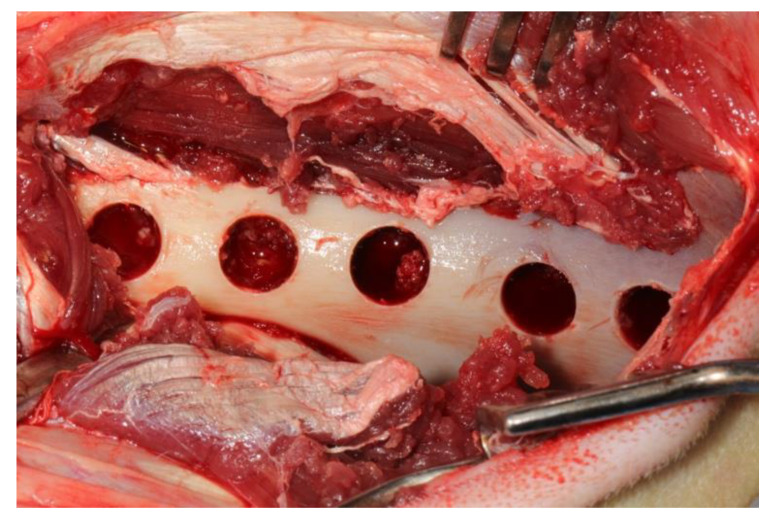
View of surgically created defects.

**Figure 3:**
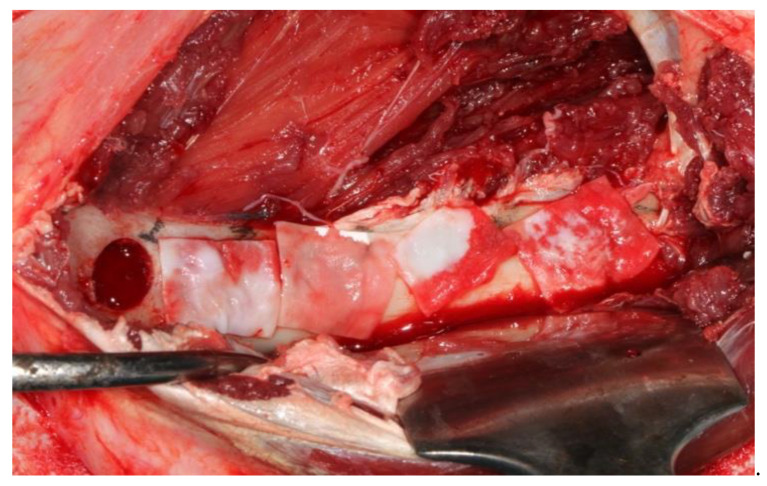
View of surgically created defects in control defects. First defect was left empty. Surfaces of the second and third defects were covered with collagen membrane after they were filled with autogenous bone particles (group G+CM). Surfaces of the fourth and fifth defects were only covered with collagen membrane as they were left unfilled (group CM).

**Figure 4:**
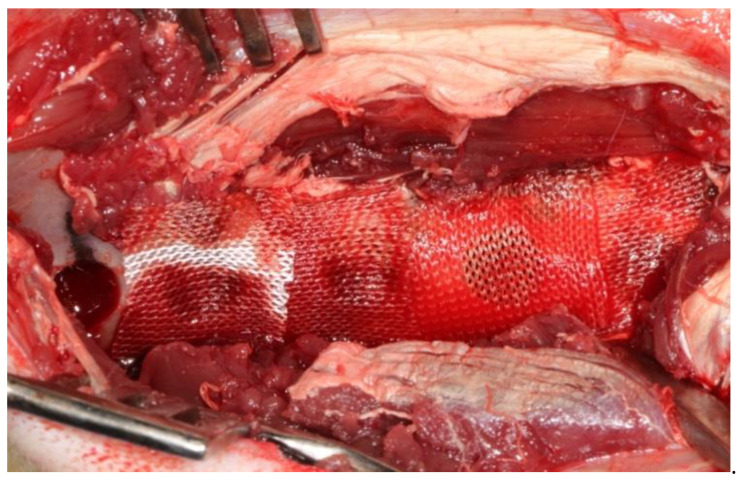
View of surgically created defects in test defects. First defect was left empty. Surfaces of the second and third defects were covered with Hyalonect membrane after they were filled with autogenous bone particles (group G+HY). Surfaces of the fourth and fifth defects were only covered with collagen membrane as they were left unfilled (group HY).

After the administration of anesthetic, the sheep were positioned sternally and both sides of the pelvis were shaved and the surgical area was sterilized with 10% povidone-iodine (Poviseptin, Mertsel Tic. A.S., Turkey). Skin and subcutaneous connective tissue were dissected following the incision parallel to the longitudinal axis of the ilium. The gluteal muscle group on the wing of the ilium was carefully dissected and then the periost was elevated with the help of a periosteal elevator. Five 10 mm diameter defects were surgically created on dorsal surfaces of both wings of the ilium. In addition, 5 mm space was reserved by using a periodontal probebetween every two defects (Figure 2). A 10 mm trephine bur was mounted on a handpiece used for implant operations to create standard defects using a physiodispenser under saline solution irrigation. Blocks of autogenous bone accumulated inside the trephine bur during the creation of defects were ground into particles using a bone grinder (Ocean System, Bursa, Turkey) to make ABG. There was no randomization in this study. Defects on the right ilium were used as the control group and on the left ilium as the test group for each experimental animal. The first defect in each animal’s right ilium was left unfilled (empty defect group, ED). Surfaces of the second and third defects were covered with collagen membrane after they were filled with autogenous bone particles (group G+CM). Surfaces of the fourth and fifth defects were only covered with collagen membrane as they were left unfilled (group CM) (Figure 3). The first defects in each animal’s left ilium were left unfilled (ED). The surfaces of the second and third defects were covered with hyaluronic acid membrane after they were filled with autogenous bone particles (group G+HY). The fourth and fifth defects were left unfilled, and their surfaces were covered only with the hyaluronic acid membrane (group HY) (Figure 4). Following the completion of the surgical procedure, the muscle tissues were restored to their previous positions, and then fasciae were sutured with monofilament resorbable suture material (Monocryl No 1, Ethicon, Johnson & Johnson, New Jersey, USA), and the skin tissue with monofilament nonresorbable polypropylene suture material (Medilen No 1, Medeks, Turkey).

### 2.4. Postoperative Care

All the experimental animals were intramuscularly administered meloxicam (0.5 mg/kg) (Bavet İlaç A.S., Istanbul, Turkey) as an analgesic and ceftriaxone sodium (2 mg/kg; 1 g, Iesef, Ulagay, Istanbul, Turkey) as an antibiotic 30 minutes prior to awakening and for 5 days following the operation. After surgeries, the animals were placed in separate cages, which had at least a 5 m^2^ shelter area. All animals were fed a standard diet. Nonresorbable sutures were removed on the 10th postoperative day.

During the study period, the sheep were examined for any complications such as leg fractures and general health status. The animals were randomly euthanized on the third and sixth weeks (three animals at each time interval). After being anesthetized with xylazine and ketamine, the animals were intravenously euthanized using 50 mg/kg of 10% sodium pentothal solution. The ilia were carefully dissected free from soft tissues, and hard-tissue samples were transferred immediately into 10% buffered formalin.

### 2.5. Histologic and Histomorphometric Evaluations

Histologic and histomorphometric examinations were performed by a single-blinded examiner (V.O.). The sample material was fixed in 10% buffered formalin for one week. Following the fixation, the whole material was decalcified in a mixture of 50% formic acid solution and 20% sodium nitrate solution. After routine tissue examination, the decalcified particles embedded in paraffin blocks were cut into 5–7 micron sections and dyed with hematoxylin eosin to observe under a light microscope (Olympus BX60, Tokyo, Japan).

The sections were examined histopathologically to evaluate inflammation, necrosis, and fibrosis. Inflammation and fibrosis were graded as follows: 0: no sign +: mild ++: moderate +++: severe. Necrosis and foreign body reaction were graded as follows: 0:no sign +: existing. Bone regeneration and residual graft materials were evaluated histomorphometrically. Four areas of each defect were determined to be imaged under a microscope and then computerized. This process was carried out by using a camera (Olympus E-330, Olympus Corporation, Tokyo, Japan) attached to the computer and the microscope. Each image was then processed by the Olympus Soft Imaging System Analysis Five software on the computer to calculate the bone regeneration and residual graft area.

### 2.6. Statistical Analysis

The methodology was reviewed by an independent statistician not previously involved in the study.

Chi-square test was used for comparison of inflammation, necrosis, and fibrosis. ANOVA, Kruskal–Wallis, Tukey, and Dunn (Bonferroni) post hoc tests were used to evaluate the differences in bone regeneration for each group. T test and Mann–Whitney U test were used to evaluate the residual graft difference. Data analyses were tested via IBM SPSS Statistics version 26.0 software bundle (IBM SPSS Statistics for Windows, Version 26.0. IBM Corp., Armonk, NY, USA). The results were observed to have a significance level of *p* < 0.05.

## 3. Results

### 3.1. Clinical Findings

The animals did not show any signs of complications or adverse events. Three of the sheep were sacrificed in the third week and another three in the sixth week, and a total of 60 defects were evaluated histologically and histomorphometrically.

### 3.2. Histologic Histomorphometric Findings

**Figure 5:**
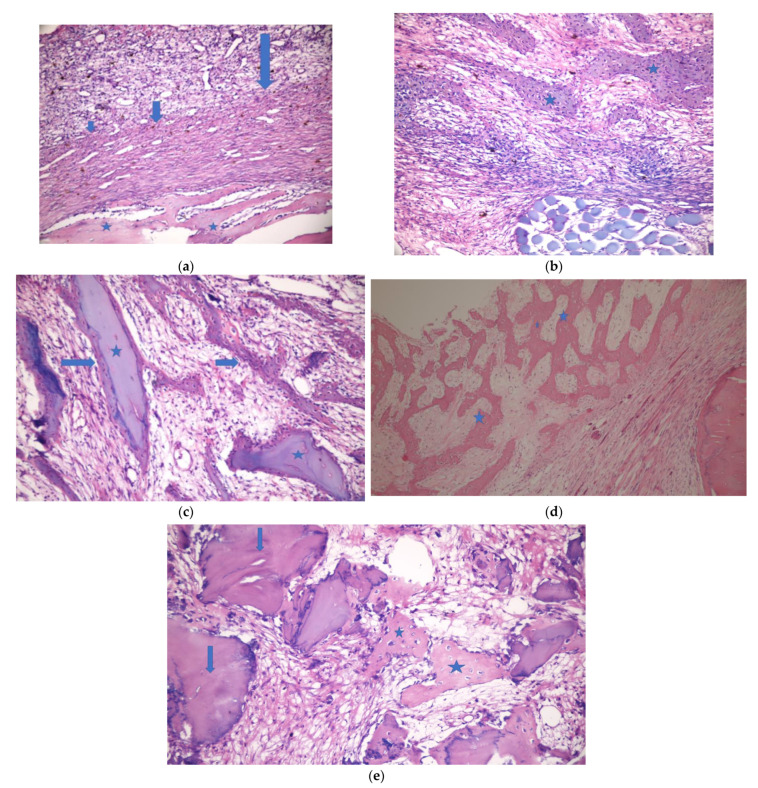
Week 3. (**a**). Empty defect group. Dense vein proliferation in the defect area and fibrosis are observed. Active osteoblasts around the bone stand out in the new bone formation areas. *: new bone formation areas; arrows: fibrosis (H&E ×200). (**b**). HY defect group. New bone formation in the defect area and hyalinized collagen tissue at the periphery are observed. *: new bone formation areas (H&E ×200). (**c**). G+HY defect group3. Bone formations surrounding the graft material are noticeable. In some areas, new bone formations surrounded by active osteoblasts inside sporadically loose collagen tissues are observed. *: graft material; arrows: new bone formation areas (H&E ×400). (**d**). CM defect group. New bone formation areas forming anastomosis inside active collagen tissue in the defect area, more fibrotic collagen tissue at the periphery. *: new bone formation (H&E ×100). (**e**). G+CM defect group. New bone formation areas and graft residues in sporadically loose collagen tissue are present. Active osteoblasts are observed around trabeculas. *: new bone formation areas; arrows: graft (H&E ×400).

**Figure 6:**
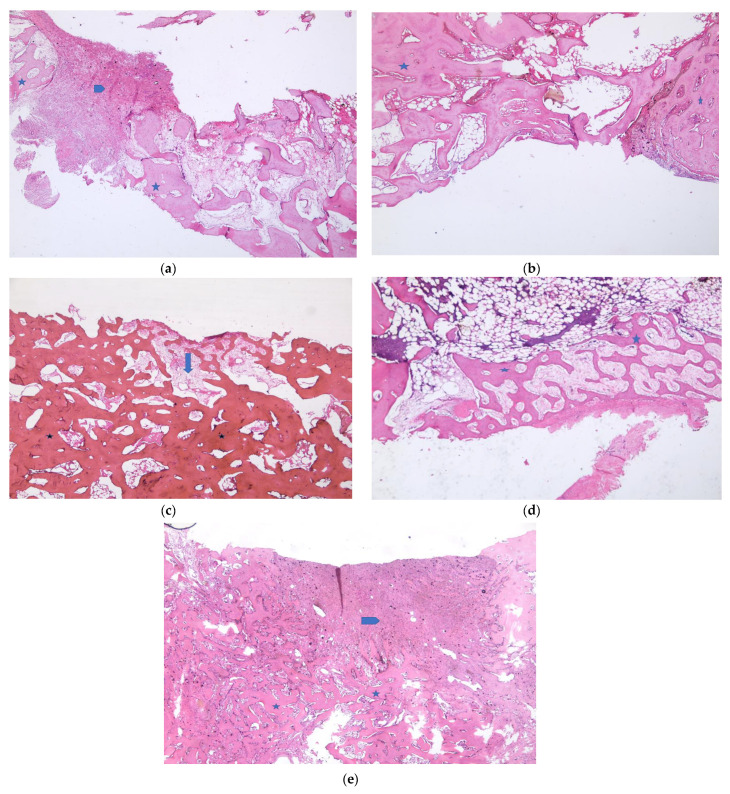
Week 6. (**a**). Empty defect group. Fibrosis and a small amount of vein proliferation among new bone formations in the defect area are observed. *: new bone formation areas; arrows: fibrosis (H&E ×40). (**b**). HY defect group. Highly mature new bone formation is observed from one end of the defect area. Fatty bone marrow is present in between. *: new bone formation areas (H&E ×200). (**c**). G+HY defect group. Sporadic fibrosis among highly dense, mature-looking bone tissues. *: new bone formation areas; arrows: fibrosis (H&E ×40). (**d**). CM defect group. New bone trabeculas connecting with each other and accompanied by loose collagen tissue that is almost filling the defect area are observed. *: new bone formation areas (H&E ×40). (**e**). G+CM defect group. Clusters of fibrosis among the dense new bone formation areas in the defect are observed. *: new bone formation areas; arrows: fibrosis (H&E ×40).

Figure 5 and Figure 6 show the histopathologic examination of the groups at the 3rd and 6th weeks.

#### 3.2.1. Inflammation

In animals sacrificed in week 3, the most significant inflammation was observed in the empty defect group of which three were classified as mild and one as moderate. In week 6, inflammation was observed in only one defect in the empty defect group, while the other defects showed no signs of inflammation. Inflammation scores for each group are listed in Table 1. No statistical significance was observed between the inflammation scores of week 3 and week 6.

#### 3.2.2. Necrosis

The evaluation of groups showed that necrosis was specifically present in the empty defect group in week 3. There was no sign of necrosis present in the CM group. In week 6, necrosis was observed in only one of the ED groups out of all groups. Table 2 shows the distribution of necrosis in the groups. There was no statistical significance present between the groups in terms of the distribution of necrosis in weeks 3 and 6.

#### 3.2.3. Fibrosis

Moderate or severe fibrosis was observed in all defects in weeks 3 and 6 but decreased in all groups in week 6. There was no statistical significance present between fibrosis scores of the groups in weeks 3 and 6. (Table 3).

#### 3.2.4. Foreign Body Reaction

There was no foreign body reaction observed in any defect in weeks 3 and 6.

#### 3.2.5. New Bone Formation

At week three: It was observed that no defect was completely filled with new bone, and the bone healing process was at the fibrous callus stage. The lowest new bone formation rate in week 3 was observed in the ED group (19.67 ± 2.73). Similar values were found for HY and CM groups (35.67 ± 4.55 ve 40.00 ± 3.41, respectively), while the highest values were observed to be in the G+CM group (52.83 ± 3.06). Statistically, the significantly highest healing rate was observed in the G+CM group. The G+CM group was observed to have a significantly higher new bone formation rate than all the other groups in week 3, followed by the G+HY group (46.33 ± 2.25). G+HY group was found to have statistically significant higher new bone formation than the ED, CM, and HY defect groups. No statistical significance was found between CM and HY groups in which only membrane without graft material was applied to defects. Additionally, the new bone formation rate in both groups was found to be significantly lower than group G+HY and G+CM in which membrane was applied in combination with graft material.

**Figure 7:**
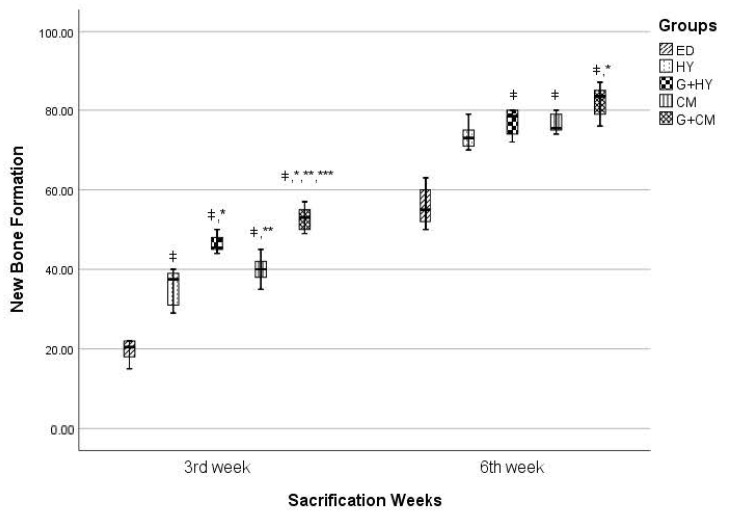
ǂ *p* < 0.05 when compared with ED. * *p* < 0.05 when compared with HY. ** *p* < 0.05 when compared with G+HY. *** *p* < 0.001 when compared with CM.

At week six: An increase in the bone fill ratio of the defects in all groups was observed in week 6. The formation of the bone bridge was found to be at different levels for each defect. Half of the defects in the ED group were observed to be full with bone tissue (55.83 ± 4.92). Almost all defects in the remaining groups were found to be full with new bone formation. Although the new bone formation in the ED groups was found to be lower than that in group HY ((73.50 ± 3.27), the difference was statistically insignificant. New bone formation in group HY was observed to be insignificantly lower than that in group G+HY and CM (*p* = 0,172, *p* = 0,285, respectively). New bone formation rates in group G+HY and CM were found to be, respectively, as follows: 77.17 ± 3.49, 76.50 ± 2.43; *p* = 0.767. The best new bone formation was observed in group G+CM ( 82.33 ± 4.08) in which almost all defects were found to be full with new bone formation. There was no difference in new bone formation rates between groups G+CM, G+HY, and CM (*p* = 0.206, *p* = 0.118, respectively) (Figure 7).

#### 3.2.6. Residual Graft

**Figure 8:**
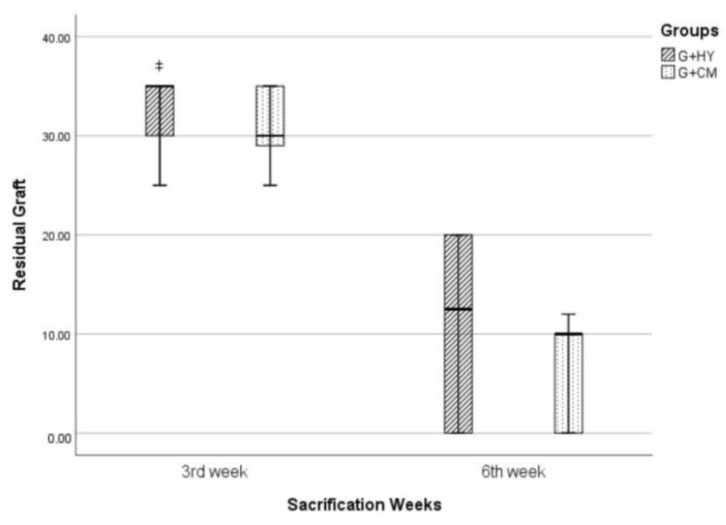
ǂ *p* < 0.001 when compared with G+CM.

In weeks 3 and 6, the residual graft was detected in groups G+CM and G+HY with defects covered with graft material. Statistically, the residual graft amount in group G+HY in week 3 was significantly higher than that in group G+CM. Although a higher amount of residual graft was observed in group G+HY in week 6, the difference was statistically insignificant (Figure 8).

## 4. Discussion

This study was planned as an experiment on sheep to evaluate the effectiveness of Hyalonect mesh or the ABG graft and Hyalonect combination on intraosseous defects. As the control group, the defects were left empty or filled and covered with ABG and CM. Statistically, the new bone formation rate in the postop third week was significantly higher in the G+CM group than all the other groups. Similarly, the defects in groups G+CM and G+HY were observed to be substantially filled with new bone formation in the sixth week. The new bone formation rates were observed to be similar in both third and sixth weeks in those groups where only CM or HY membrane was applied.

Hyalonect membrane was produced to be used in the field of orthopedics based on HA’s effectiveness on wound healing, making orthopedic studies being the main field to evaluate the success of Hyalonect use. Rhodes et al. [13] removed dorsal muscular fascia of 6 mm radius and 1 mm deep in 162 rats and then sutured Hyalonect membrane onto this defect area in the experimental group to evaluate Hyalonect’s biological characteristics. A defect of the same size was prepared but not covered with the membrane in the control group. Ten rats at a time in the experimental group and eight in the control group were sacrificed and evaluated histologically on days 15, 30, 60, 90, 120, 180, 270, 365, and 540. It was observed that cellular colonization occurred on day 30, vascularization on day 120, matrix fiber organization on day 270, and similarity of connective tissue to surrounding tissues on day 365 and 540 in the experimental group. Macroscopic examination revealed no signs of edema, serous formation, inflammation, infection, or wound-site inflammation. Histological examination of soft tissue was not performed in our study. However, no opening was present in the soft tissue on the surface of the grafted area. Another study was conducted in dogs by the same research team to evaluate the direct membrane effect of the Hyalonect membrane in the cases of graft applied bone defects where the host membrane did not provide sufficient covering [13]. Demineralized bone matrix, plain putty, allomatrix, or autogenous graft was applied to the defects of 9 mm radius, 5 mm deep in the proximal humeri of seven dogs, and then the graft surfaces were covered with Hyalonect membrane. Histological evaluation was performed following the sacrification of the animals after 6 weeks. The surfaces of all defects were observed to be covered with a dense and thick layer of fibrosis membrane. Hyalonect membrane was present in all specimens after 6 weeks despite the degradation to some extent. There was no overflown graft material in any defect. Resorption time of Hyalonect membrane is reported to be 40 days by the manufacturer, whereas it is 4–6 months for collagen membrane. The sacrification times were determined to be third and sixth weeks as the recovery time for the sheep is significantly shorter than human beings. It could be interpreted that the sacrification times were proper as no membrane residues were observed during sacrification and in histological samples. However, sacrification could be performed earlier (e.g., after 1 week) on sheep or different sacrification times could be determined for smaller experimental animals (e.g., rats) in order to evaluate the effect of the Hyalocent membrane on the early stage of bone healing.

Ayanoğlu et al. [19] conducted a study on the combined use of Hyalonect membrane and allograft. They prepared metaphyseal defects in the tibia of 80 rabbits. The rabbits were randomly separated into four groups as follows: group I, empty defect group; group II, Hyalonect membrane applied bone defects; group III, allograft applied defects; group IV, Hyalonect covered, allograft applied defects. Histological and radiological examinations were performed in the third and sixth weeks. Results indicated that the healing process was accelerated by the application of the Hyalonect membrane alone or with graft. Radiologically better healing was observed in group IV, compared to group III. A statistically significant healing process was observed in groups II, III, and IV, compared to group I in the third week. Group IV was observed to have a better healing process, compared to group II, in both the third and sixth weeks, and only the empty defect group in the sixth week. This study was conducted to evaluate the success of the use of Hyalonect membrane with autogenous graft in the treatment of intraosseous defects. In the third and sixth weeks, higher amounts of new bone formation were detected in all graft-applied defects, compared to those that were not applied. The type of the applied graft material might affect the healing process. The use of the Hyalonect membrane with other graft materials might result in compatibility issues. Further studies are needed to assess the effectiveness of the use of Hyalonect membrane with different graft materials.

Mermerkaya et al. [20] conducted a study to evaluate the membrane effectiveness of Hyalonect. They prepared defects in the tibial diaphysis of New Zealand albino rabbits by resecting 10 mm segments. The particles were then fixed with Kirschner wire after resection and the blood aspirated from iliac wings was administered to the defect area as a mesenchymal cell source. The surface of the defects was covered with Hyalonect membrane in the experimental group or left empty in the control group. The osteoblastic activity was evaluated in the postop fourth week by bone scintigraphy studies. The mean count/pixel ratio on the bone defect area in the Hyalonect group was significantly higher than the control group. Collagen membrane and Hyalonect membrane were used as membrane materials in our study. It indicates that both CM and HY have similar effects on empty defects as there was no difference in regard to inflammation, necrosis, fibrosis, and new bone formation between the groups with empty defects covered with CM or HY membranes in the third and sixth weeks. Although there was no difference between the ED group and HY and CM groups in regard to inflammation, necrosis, and fibrosis, it was observed that covering the surface of the defects with membrane rather than leaving them empty increased the amount of new bone formation.

It is a possible complication that openings might form on the membrane surface resulting in infection during GBR operations in the clinic. Although it is unclear why this complication develops, it might be due to the strain caused by the closing of soft tissue or the lack of vascular supply. No openings around the incision area or infection were observed both in CM and HY groups in this study. It could prove useful to evaluate the relation between Hyalonect membrane use in the clinic during GBR operation and the infection or opening in the defect area.

In preclinical studies, animal models are used to test new biomaterials. Histomorphometry is well suited for preclinical animal models that can provide substantial histological information and evaluate standardized groups [21]. In this study, sheep were used as experimental animals due to them being frequently used models in dental studies to evaluate bone healing, having a bone structure and remodeling that are similar to those of human beings, and having proper structure for wide defects [22,23,24]. Using the sheep’s right ilium as the control group and left ilium as the experimental group made it possible to host both groups on the same animal. The wide anatomical structure of the sheep ilium provided easy access to the surgical area and allowed the creation of five defects in the same ilium. None of the experimental animals had bone fractures. The lowest new bone formation rate in the empty defects, i.e., 19.67% ± 2.73 in the third week and 55.83% ± 4.92 in the sixth week, in comparison to other groups, indicates that 10 mm defect volume was adequate.

## 5. Conclusions

GBR is a frequently used surgical operation prior to dental implant application for cases with inadequate bone volume. When all the GBR operations to date are considered, it is observed that different graft materials and membrane combinations were used, but the ideal combination has yet to be defined. Therefore, active studies for different biomaterials continue. To the best of our knowledge, this is the first experimental study evaluating the feasibility of the Hyalonect membrane in GBR that was produced to provide periosteal regeneration. The results of the study indicate that Hyalonect has an effect close to that of the collagen membrane. It would be beneficial to use Hyalonect in combination with different graft materials with different experimental models. Additionally, we believe it would be useful to evaluate the clinical use of the Hyalonect membrane in GBR in future studies.

## Figures and Tables

**Table 1:** Inflammation scores in 3rd and 6th weeks. A chi-square test was used.

		Week 3		Week 6	
ED	Negative	2	33.33%	5	83.33%
	(+)	3	50.00%	1	16.67%
	(++)	1	16.67%	0	0.00%
HY	Negative	4	66.67%	6	100.00%
	(+)	1	16.67%	0	0.00%
	(++)	1	16.67%	0	0.00%
G+HY	Negative	3	50.00%	6	100.00%
	(+)	2	33.33%	0	0.00%
	(++)	1	16.67%	0	0.00%
CM	Negative	5	83.33%	6	100.00%
	(+)	1	16.67%	0	0.00%
G+CM	Negative	5	83.33%	6	100.00%
	(++)	1	16.67%	0	0.00%
*p*+		0.591		0.388	

+ = mild inflammation; ++ = moderate inflammation.

**Table 2:** Necrosis scores in the 3rd and 6th weeks. A chi-square test was used.

Necrosis		Week 3		Week 6	
ED	Negative	2	33.33%	5	83.33%
	(+)	4	66.67%	1	16.67%
HY	Negative	5	83.33%	6	100.00%
	(+)	1	16.67%	0	0.00%
G+HY	Negative	5	83.33%	6	100.00%
	(+)	1	16.67%	0	0.00%
CM	Negative	6	100.00%	6	100.00%
G+CM	Negative	5	83.33%	6	100.00%
	(+)	1	16.67%	0	0.00%
*p* +		0.073		0.388	

**Table 3:** Fibrosis scores in the 3rd and 6th weeks. A chi-square test was used.

Fibrosis		Week 3		Week 6	
ED	(+)	0	0.00%	4	66.67%
	(++)	4	66.67%	2	33.33%
	(+++)	2	33.33%	0	0.00%
HY	Negative	0	0.00%	4	66.67%
	(+)	0	0.00%	2	33.33%
	(++)	5	83.33%	0	0.00%
	(+++)	1	16.67%	0	0.00%
G+HY	Negative	0	0.00%	4	66.67%
	(+)	0	0.00%	2	33.33%
	(++)	3	50.00%	0	0.00%
	(+++)	3	50.00%	0	0.00%
CM	Negative	0	0.00%	3	50.00%
	(+)	0	0.00%	2	33.33%
	(++)	3	50.00%	0	0.00%
	(+++)	3	50.00%	1	16.67%
G+CM	Negative	0	0.00%	2	33.33%
	(+)	0	0.00%	2	33.33%
	(++)	5	83.33%	1	16.67%
	(+++)	1	16.67%	1	16.67%
*p* +		0.588		0.302	

+ = mild; ++ = moderate; +++ = severe.
